# Supplementary material for: Slowly Making Sense: A Review of the Two-Step Venom System within Slow (Nycticebus spp.) and Pygmy Lorises (Xanthonycticebus spp.)
Source: Toxins (Basel). 2023 Aug 22;15(9):514. doi: 10.3390/toxins15090514 (PMC10536643; doi:10.3390/toxins15090514)
Supplement: Supplementary file 1 [file toxins-15-00514-s001.zip › Supplementary File S3 - Research Agenda.pdf]

Slowly making sense: a review of the two-step venom system within slow (Nycticebus spp.) and pygmy lorises (Xantho-nycticebus spp.)

Leah Lucy Joscelyne Fitzpatrick, Rodrigo Ligabue-Braun and K. Anne-Isola Nekaris

A non-exhaustive list of research topics to further explore relating to the venom system of the slow loris. We have split these into research actions (i.e. an experiment or analysis that should be carried out to provide data) or research questions (i.e. hypothesis or theories surrounding these categories that may have multiple different experimental ways of approaching them). Please note that research actions and questions in each category are not necessarily linked.		
**Category**	**Research actions**	**Research questions**
Overall venom	Perform experiments that would allow full documentation and measurement of slow loris saliva, BGE, BGEsp and venom mixture on:<br>- cells (human, cancerous and noncancerous)<br>-Invertebrate animal models<br>-Mammal animal models	What are the effects of slow loris secretions on human cells?
	Identify the LD50 of slow loris venom on both invertebrate and vertebrate models	What are the effects of slow loris secretions on invertebrates?
	Measure exactly the quantities of both saliva and brachial gland produced by different species of slow loris	What are the effects of slow loris secretions on mammals?
Specific components of venom activation	Isolation of C1R from *N. javanicus* saliva and applied to both cell, invertebrate and mammal animal models to measure effects	What response does the slow loris immune system have to be bitten by another slow loris in intraspecific competition? Do we see in response in blood? Urine? Saliva?
	Isolation of BGEsp from any slow loris species and applied to both cell, invertebrate and mammal animal models to measure effects	What response does the human immune system have to be bitten by a slow loris? Do we see in response in blood? Urine? Saliva?

Saliva	Full updated list of all secretions from the saliva of slow lorises	Does the composition of slow loris saliva vary by species, age, sex, season or captive status?
	Saliva samples obtained and analysed from all slow loris species	How does the composition of slow loris saliva compare to slender lorises?
	Full nucleotide and protein sequence of C1R taken from *N. javanicus* saliva	Are there any other immunity-based proteins or products expressed within the saliva gland such as a CVF like protein?
Brachial Gland	Full updated documented list of all proteins secreted in the brachial gland of slow lorises	Do the components of the brachial gland vary by species, age, sex, season or captive status?
	Brachial gland secretions obtained and analysed from all slow loris species	How is the composition of the brachial gland in slow lorises compared to slender lorises?
		Does diet influence the composition of the brachial gland exudate?
BGEsp	Confirm presence of BGEsp within all slow loris species	Does the BGEsp vary between slow loris species, age, sex, season or captive status?
	Obtain full nucleotide, amino acid sequence, and glycosylation profile of the BGEsp	Do different glycosylation models change how the protein is formed?
	Obtain full nucleotide and amino acid sequence of BGEsp from each slow loris species	Is BGEsp affected by different chemicals from trees consumed by slow lorises?
		How does the shape of BGEsp change when added to solvents that dissolve either hydrophilic compounds (i.e. FA) or lipids (ie MM)?
Diet and Exudate	Further identification of trees and plant matter that slow lorises consume in the wild and toxic insects	Do slow lorises in captivity have a preference for exudates from their native range over available commercial products? (Strong observational support available)
	Identification to genus or species level on invertebrates that slow lorises consume in the wild	Are there any measurable effects on slow loris saliva (i.e., pH) when consuming diets with exudates from potential cytotoxic trees?
		Does diet change the composition of the brachial gland?
		Does diet change the composition of saliva?
		Are there any chemicals or products that are likely to interact with the BGEsp? If so, what change would we expect to see?

Behaviour (slow lorises)	Ethogram produced for all slow loris species (based on Javan slow loris one)	Do all slow loris species engage in intraspecific competition using BGE?
		How do slow lorises react to other individuals BGE?
		Are the percentage of bites wounds in slow lorises of similar levels to those documented in *Xanthonycticebus spp., N. javanicus* or *N. coucang*?
		Are trauma levels similar in rescue centres across Asia equivalent to what has been documented in captive settings so far?
		Are trauma levels similar in zoos across the globe to that seen in North American zoos in 1980-2010?
Behaviour (predators)	A full collation of all observations of predation events upon a slow loris	What reactions do snakes produce when exposed to BGE, slow loris saliva and the venom combination?
		Do potential/confirmed predators of slow lorises have similar reactions to slow lorises and cobras?
		How do predators react to saliva, BGE and both combined into venom? Similar to different reactions?
		Do predators have different reactions to BGE depending on sex, age, species, season and captive status of the slow loris?
Evolution		Which venomous species of snake (particularly *Naja spp.*) overlap with the slow lorises range? Is that shared by the slender loris?
		Using palaeontology, geological and distribution, can we predict when slow lorises distributed across SEA, especially across the islands?

Genomic	Generate multiple high chromosome level drafts of all other loris species (Currently only *Xanthonycticebus spp.*, *N.bengalensis* and *N.coucang* exist)	Are olfactory related genes such as Vomeronasal type 1 receptor (*V1R*) or olfactory receptor genes (*ORG*) upregulated or expanded within slow loris genomes?
		Are genes in the secretoglobin family upregulated or expanded within slow loris genomes?
		Are any genes involved within the immune system (and specifically genes involved with the complement system) upregulated or expanded within slow loris genome?
		Does a syntenic comparison between other strepsirrhine primate genomes identify which genes have been adapted and changed specifically within Lorisinae and slow lorises?
Transcriptomic	Obtain transcriptomics of saliva glands for any/ all slow loris species	Is there evidence of post translational modification of saliva products that could be related to venom?
	Obtain transcriptomics of brachial gland for any/all slow loris species	Is there evidence of post translational modification of brachial gland products that could be related to venom?
